# Social-Ecological Analysis of the Factors Influencing Shanghai Adolescents’ Table Tennis Skills: A Cross-Sectional Study

**DOI:** 10.3389/fpsyg.2020.01372

**Published:** 2020-06-30

**Authors:** Yi Xiao, Wenwen Huang, Miaomiao Lu, Xiaoling Ren, Pei Zhang

**Affiliations:** ^1^China Table Tennis College, Shanghai University of Sport, Shanghai, China; ^2^School of Economics and Management, Shanghai University of Sport, Shanghai, China

**Keywords:** social support, self-efficacy, adolescents, physical activity, table tennis skills

## Abstract

The main purpose of this study was to explore the factors that affect adolescents’ table tennis skills (ATTS) among adolescents in Shanghai from the social ecological perspective, including individual factors, social support, and physical environment. 1,526 students from Shanghai primary and secondary schools were included in this study (age = 12.31 ± 1.32 years). Participants completed a questionnaire based on social ecological theory after taking the ATTS test. A structural equation model was applied to test the relationships among the study variables. The relationship model incorporating individual factors, social support, physical environment, and ATTS test score fit the data well (χ^2^ = 1415.754, χ^2^/df = 4.96, *p* < 0.001; CFI = 0.914; IFI = 0.930; NFI = 0.921; RMSEA = 0.032). The investigation results showed that social support and physical environment promote adolescents’ scores on the ATTS test. At all levels of social ecology, individual factors were the most important factors for the improvement of ATTS. The level impacting most significantly on ATTS was individual factors, followed by social support, and, finally, the physical environment. Therefore, cultivating intrinsic interest is an important way to facilitate the continuous activities of adolescents. In addition, friends should support each other, and parents should give adolescents appropriate encouragement regarding table tennis exercise. Schools should provide more table tennis facilities. PE teachers should respect adolescents’ ideas, listen to students’ opinions, and encourage them to participate in table tennis training.

## Introduction

Research has demonstrated that playing table tennis can increase adolescents’ physical fitness, relieve learning pressure, prevent and improve myopia, enhance the body’s sensitivity and coordination, promote learning efficiency and communication with other students, and cultivate adolescents’ psychokinesis (He, 2012; Liu and Li, 2013). However, the number of minors taking part in China’s table tennis is declining year by year, and only 10% of students have been contacted with table tennis regularly (Yan, 2014). The reasons are as follows: adolescents’ heavy study pressure; the increasing influence of other sports, such as badminton, in recent years; and adolescents’ poor table tennis skills (Zhou et al., 2018; Qin, 2019). All of the above-mentioned factors lead to adolescents’ lack of interest in table tennis. Therefore, possessing certain sport skills is necessary for cultivating adolescents’ sports interest and adhering to life-long physical exercise (Wang, 2012).

The formation of ATTS not only requires adolescents to actively participate in table tennis and carefully practice basic table tennis skills under a PE teacher’s guidance, but also is related to other individual factors such as age, sex, and previous sports experiences (Faber et al., 2016). Internal factors are the main factors that affect adolescents’ table tennis participation, and external factors are also important (Shao, 2015; Condello et al., 2016). Dealing with the relationship between internal factors and external factors well can help adolescents better master table tennis skills (Faber et al., 2016). At present, the research on the teaching of table tennis in primary and middle schools mainly focuses on teaching methods, teaching content, and effect evaluation (Chen, 2015; Yu, 2016). The existing research is not deep enough and has not been extended to the specific research on the influencing factors of table tennis skills.

In addition, good table tennis skills are necessary for regular table tennis exercise participation, and regular table tennis exercise participation helps in enhancing table tennis sport skills in return (Faber et al., 2016). Table tennis skills test score was used to reflect adolescents’ table tennis sport skills level and their table tennis exercise participation (China Table Tennis College, 2017). The implementation of the ATTS test can enrich daily table tennis activities, facilitate the mastery of table tennis skills, and improve adolescents’ physical and mental health.

Social ecology investigates the relationship between an individual and the environment. In the context of health promotion, the socio-ecological framework emphasizes the influence of individual factors, social support, and physical environment on activity patterns, such as adequate sports facilities, verbal encouragement of teachers and friends, and physical participation of parents, all of which have an impact on individual physical activity (PA) (Golden and Earp, 2012).

Individual factors in this research included two parts: self-efficacy and motivation. Self-efficacy represents “trust [in] one’s ability to organize and execute actions that can achieve a certain achievement” and has been always associated with adolescents’ PA (Lee and Young, 2018). There are striking and positive relationships between self-efficacy and adolescents’ initiation, participation, and self-regulation in PA (Cortis et al., 2017). Self-efficacy is a determinant of motivating new PA behaviors, how long individuals will persist when facing negative experiences, and how much effort they will put into physical activities (Bandura et al., 1999). Self-efficacy is of great importance in the adoption and the maintenance of different periods of exercise behavior (McAuley et al., 2003).

There are three types of motivation, named intrinsic motivation, extrinsic motivation, and amotivation, to explain the different reasons why individuals engage in activities (Deci and Ryan, 2004). Intrinsic motivation was identified as the spontaneous engagement in activities for their own sake, such as the feelings of pleasure, interest, and satisfaction derived directly from participation. Extrinsic motivation, as opposed to intrinsic motivation, drives the participation of an individual in activities through external stimuli (e.g., threat, reward, punishment). Amotivation, stemming from a lack of competence, is the belief that an activity is unimportant, and/or when an individual does not perceive contingencies between her/his behavior and the desired outcome(s). Motivation is a major determinant of the issue on sports interest (Kondric et al., 2013).

Social support was identified as a source of motivation by many of the participants who were adult cancer survivors (Barber, 2013). A growing volume of literature has reported significant correlations between self-efficacy, social support, and PA in adults as well as other age groups (Lee and Young, 2018). More exercise social support leads to a stronger sense of exercise self-efficacy and more positive affective responses to exercise participation when a program is finished (Hagger et al., 2002; Wiltshire et al., 2017). Receiving encouragement from significant others and having a companion for PA were associated with higher PA in children and adolescents (Jaeschke et al., 2017). Peers and PE teachers played an extremely important role in generating PA behavior; weekly observations of PE classes have shown that more positive behavior can be inspired from PE teachers’ interest and positive reinforcement, which is the same as small “cliques” (Daigle, 2003; Barber, 2013).

A previous study has indicated that children’s participation in PA and environmental attributes were correlated (Davison and Lawson, 2006). The availability of physical exercise programs and equipment in schools and neighborhood characteristics such as pedestrian and cyclist safety structures were positively correlated with physical exercise for children and adolescents (Carlin et al., 2017). A community with more PA resources nearby may offer residents more opportunities to exercise with friends, and both neighborhood safety and device accessibility had significant correlations with PA in a population-based adolescent sample (Molnar et al., 2004; D’Angelo et al., 2017). School also plays an extremely important role in all levels for adolescents’ PA (Ip et al., 2017).

In China, studies on ATTS are mainly from the perspective of pedagogy but rarely from the perspective of social ecology. Based on the perspective of social ecology, this study explored the factors that affect ATTS and built a social–ecological model to improve Shanghai ATTS. To learn the reasons for adolescents’ poor skills and low participation in table tennis, this study analyzed the multiple factors affecting table tennis skills and put forward suggestions for further improving ATTS among Shanghai adolescents and promoting the popularization of table tennis among adolescents. Thus, this study contributes to the improvement of the ecological environment of adolescents’ table tennis in Shanghai and the promotion of Shanghai adolescents’ physical health.

It was hypothesized that: (1) individual factors (motivation, self-efficacy), social support (parent support, friend support, PE teacher support), and physical environment (school environment, community environment) have an impact on the performance of the ATTS test (Xiang, 2019); (2) among the levels of social ecology, different levels have different degrees of impact on the improvement of ATTS (Xiang, 2019); and (3) in addition to the direct effect, the influence of individual factors on the performance of the ATTS test has a mediating effect. Social support and physical environment can indirectly influence the performance of the ATTS level test through individual factors.

## Materials and Methods

### Participants

The study was approved by the university ethics committee in Shanghai. In this study, table tennis skills test score was used to reflect adolescents’ table tennis sport skills level and their table tennis exercise participation. Participants were randomly recruited from 12 districts of Shanghai, with one primary and one secondary school per district, and about 70 students from each school, using the stratified sampling method. The sampling inclusion criteria were as follows: primary or secondary school students (ranging from grades 3 to 9), agreeing to take the ATTS test, and Shanghai residents. Finally, 1,823 students who met the inclusion criteria were invited to attend the study. Written consent forms were distributed to all 1,823 students and their parents prior to data collection. A total of 1,628 students from 12 districts agreed to participate in this study.

A total of 1,576 participants out of the 1,628 students voluntarily returned the survey. Of the 1,576 participants, 50 participants were subsequently excluded because their response to the questionnaires was not complete. The final analytic sample consisted of 1,526 students whose ages ranged from 9 to 16 years old (age = 12.31 ± 1.32 years). A total of 755 (49.48%) and 771 (50.52%) were primary and secondary students, respectively.

### Procedure

Before data collection, the participants were given a full explanation about the purpose of the study, the potential benefits/risks, and their confidentiality and withdrawal rights. Then, they were directed to China Table Tennis College training hall to attend the ATTS test. After finishing the ATTS test, they were directed to complete the self-reported questionnaires. For participants under 16 years old, questionnaires were completed with the help of their parents. Participants spent approximately 20–30 min to complete the questionnaires.

### Measures

#### Demographics

Self-reported personal information on age, sex, residential region, frequency, duration, and intensity of table tennis activities was obtained from the questionnaires.

There were four programs tested through exercises in ATTS evaluation. ATTS test score = a^∗^0.25 + b^∗^2^∗^0.25 + c + 25/40^∗^d (National Youth Sports Skills Standards Development Group, 2017). Here, a represents the valid number of times the ball was bounced on the racket, b represents the valid number of times the ball was bounced against the wall, c represents the score of balancing the ball while walking around the table, and d represents the number of times a ball was caught when the serving machine served 40 balls to the participant.

#### Individual Factors

##### Self-efficacy

Adolescents’ self-efficacy was assessed by an eight-item scale. The content of this scale pertained to students’ confidence in their ability to be physically active (Motl et al., 2005). Each item was rated on a five-point Likert-type response scale. Mean was obtained from each item. The internal consistency of efficacy measurements based on the Cronbach coefficient α was 0.853.

##### Motivation

The scale used to assess different types of motivation was developed by Goudas et al. (1994). Five subscales need to be answered by participants, which were Intrinsic Motivation, Identified Rules, Introjected Regulation, External Regulations, and Amotivation (Standage et al., 2005). Mean score was obtained from each item, and the Cronbach coefficient α was 0.887.

#### Social Support

Social support in this study included three dimensions: parent support, friend support, and PE teacher support.

##### Social support from parents

A parental support scale was developed for Amherst Health and Activity Research. Adolescents and assessed parents completed five parental support scales together (Prochaska et al., 2002). Each item was rated on a five-point Likert-type response scale. Those responses were ranging from 1 (strongly disagree) to 5 (strongly agree), and then the mean score of all items was calculated to represent that scale. The Cronbach coefficient α was 0.927.

##### Social support from friends

The four-item friend support scale was developed by Prochaska et al. (2002). The scale included the following: encourage, participate, praise, and encourage friends to participate. Each item was rated on a five-point Likert-type scale ranging from 1 (strongly disagree) to 5 (strongly agree), and then the mean score of all items was calculated. The Cronbach coefficient α was 0.868.

##### Social support from PE teachers

A six-item scale was used to assess perceived social support about PE teachers. The scale’s response options were ranging from 1 (strongly disagree) to 5 (strongly agree) (Daigle, 2003). There were two items included in the stem “In my PE class” to indicate the teacher support: “my teacher really listens to what I want to say” and “my teacher encourages me to do the best I can.” Finally, the mean score was obtained from each item. The Cronbach coefficient α was 0.892.

#### Physical Environment

The method of environmental assessment was to quantify the extent to which the environment contains resources that promote or hinder PA.

##### Community environment

A scale developed by Motl et al. (2005) was used to evaluate equipment accessibility and perceived safety. The metric for perceived environment consisted of four items, each with a score range of 5, from 1 (strongly disagree) to 5 (strongly agree). The means of these items were used as an overall indication of the magnitude of equipment accessibility and perceived safety. The Cronbach coefficient α was 0.797.

##### School environment

Robertson-Wilson et al. (2007) developed 12 items to assess the school’s physical environment. Each item was rated on a five-point Likert-type scale ranging from 1 (strongly disagree) to 5 (strongly agree), and the means of these items were used as an overall indication of the magnitude of school PA environment. The Cronbach coefficient α was 0.890.

The Chinese versions of those scales were translated back into English to test language validity. The Chinese scales had been found to exhibit a good value for reliability and validity and had been widely used in Chinese populations.

### Data Analysis

Date analysis was performed using SPSS 22.0. Participants’ demographic variables were summarized using descriptive statistics. Internal consistency reliability for each scale was assessed by Cronbach’s alpha. Pearson correlation matrix analyses were employed to explore the relationship among social support, individual factors, physical environment, and the ATTS test score. Hierarchical regressions were conducted to separately examine the strength of associations of social support, individual factors, and physical environment with the ATTS test score. To test the *R*^2^ change at each step, individual factors, including self-efficacy and motivation, were entered first; social support from parents, friends, and PE teachers was entered in the second block; and the school and community environmental factors were entered in the third block (Gu et al., 2014). Independent-samples *T*-test was used to analyze the effect of different age and sex on the ATTS test score. ANOVA was applied to examine the effect of the frequency of participating in table tennis per week and the duration of each table tennis exercise on the ATTS test score. Structural equation modeling (SEM) was employed to check the proposed model and to test the impact of individual factors, social support, and physical environmental variables on the ATTS test scores. Various indices for model-data fit, including the χ^2^, CFI, RFI, IFI, NFI, and RMSEA were used to assess the model fit to the data. The alpha level in the multiple regression analyses was 0.05. The SEM was assessed using AMOS 22.0.

## Results

The ATTS test score was positively related to motivation, self-efficacy, social support from parents, friends, and PE teachers, community environment, and school environment (*r*’s ranging from 0.490 to 0.862, all *p* < 0.01) (Table 1). Among all study variables, motivation, social support from friends, and self-efficacy were highly correlated with the ATTS test score (*r* = 0.862, *r* = 0.852, and *r* = 0.829, respectively). Among individual factor variables, motivation had a higher correlation with the ATTS test score than did self-efficacy (*r* = 0.862 and *r* = 0.829, respectively). Among social support variables, friends’ support showed greater correlation with the ATTS test score than parents’ support, and the weakest factor was PE teachers’ support (*r* = 0.852, *r* = 0.739, and *r* = 0.650, respectively). Among physical environment variables, the effect of school environment on the ATTS test score was higher than that of community environment (*r* = 0.590 and *r* = 0.490, respectively). In addition, self-efficacy and motivation were positively related to social support and physical environment (*r*’s ranging from 0.428 to 0.788 and from 0.368 to 708, respectively, all *p* < 0.01). Furthermore, self-efficacy was positively correlated with motivation (*r* = 0.684, *p* < 0.01).

**TABLE 1:** Internal consistency reliabilities and correlations among variables (*N* = 1,526).

Variables	1	2	3	4	5	6	7	8
1. ATTS test score	–							
2. PE teachers’ support	0.650**	(0.892)						
3. Friends’ support	0.852**	0.499**	(0.868)					
4. Parents’ support	0.739**	0.423**	0.759**	(0.927)				
5. Community environment	0.490**	0.253**	0.480**	0.464**	(0.797)			
6. School environment	0.590**	0.451**	0.388**	0.319**	0.210**	(0.890)		
7. Self-efficacy	0.829**	0.578**	0.788**	0.769**	0.522**	0.428**	(0.853)	
8. Motivation	0.862**	0.660**	0.708**	0.591**	0.368**	0.569**	0.684**	(0.887)

*Note.* Cronbach’s alpha coefficients are provided along the diagonal; several internal consistency alphas are not possible due to a single-item scale. **p < 0.01.

The mean scores of self-efficacy, motivation, social support from friends, parents, and PE teachers, community environment, and school environment exceeded the midpoint of the scales, showing positive perceptions. Furthermore, most of the participants had higher self-efficacy and more social support from friends and PE teachers (Table 2).

**TABLE 2:** Descriptive statistics for independent and dependent variables (*N* = 1,526).

Variables	Minimum	Maximum	Mean	SD
ATTS test score (pt)	11.88	99.38	72.74	16.62
PE teachers’ support	1.00	5.00	3.50	0.87
Friends’ support	1.00	5.00	3.56	1.04
Parents’ support	1.00	5.00	3.36	1.02
Community environment	1.00	5.00	3.04	0.88
School environment	1.00	5.00	3.09	0.88
Self-efficacy	1.00	5.00	3.57	0.80
Motivation	1.00	5.00	3.45	1.08

The result of hierarchical regression is shown in Table 3. Individual factors, including self-efficacy (β = 0.450, *p* < 0.01) and motivation (β = 0.554, *p* < 0.01), accounted for 85.1% of the variance in the test score (*R*^2^ = 0.851, *F* = 4,332.673, *p* < 0.01). When social support factors (from friends, parents, and PE teachers) were entered in the second block, the model accounted for an additional 3.8% of the variance (*R*^2^ = 0.889, *F* = 173.976, *p* < 0.01). The effects of individual factors declined (β = 0.209 and β = 0.412, respectively), and support from friends (β = 0.301, *p* < 0.01) made a greater contribution to the ATTS test score than self-efficacy (β = 0.209, *p* < 0.01) did, while this effect was weaker than motivation (β = 0.412, *p* < 0.01). Supports from PE teachers and parents made a little contribution to the ATTS test score (β = 0.075, *p* < 0.01). All individual factors and social support factors emerged as significant indicators of participants’ ATTS test scores. When physical environmental factors were entered in the third block, the model accounted for an additional 1.4% of the variance. School environment (β = 0.140, *p* < 0.01) contributed to the reported test score more than community environment (β = 0.045, *p* < 0.01) did. Friends’ support (β = 0.304, *p* < 0.01) and motivation (β = 0.342, *p* < 0.01) made greater contributions to the ATTS score than other social ecological factors did. The final full model accounted for 90.3% of the variance in participants’ test score (*R*^2^ = 0.903, *F* = 111.806, *p* < 0.01). All individual factors, social support factors, and physical environmental factors were significant correlates of participants’ ATTS test scores.

**TABLE 3:** Hierarchical regression of social ecological factors on test score (*N* = 1,526).

Step#	Independent variables	*R*^2^	*R*^2^ change	β	*F* change
Step 1	Individual factors	0.851	0.851		4,332.673**
	Self-efficacy			0.450**	
	Motivation			0.554**	
Step 2	Social support factors	0.889	0.038		173.976**
	Self-efficacy			0.209**	
	Motivation			0.412**	
	PE teachers’ support			0.075**	
	Friends’ support			0.301**	
	Parents’ support			0.075**	
Step 3	Physical environmental	0.903	0.014		111.806**
	Self-efficacy			0.173**	
	Motivation			0.342**	
	PE teachers’ support			0.063**	
	Friends’ support			0.304**	
	Parents’ support			0.081**	
	Community environment			0.045**	
	School environment			0.140**	

Note. R^2^ values are cumulative, with each incremental step adding to the variance explained; β values are standardized regression coefficients at each step of the regression analysis. **p < 0.01. All VIF values of independent variables for each regression model were less than 5.

The result of independent-samples *T*-test indicated that different sex had significant differences in the ATTS test score (*p* < 0.01), and the male’s test score was higher than the female’s. Besides, different ages (9–11 years old, 12–16 years old) also had significant differences in the ATTS test score (*p* < 0.01), and the participants’ age from 12 to 16 years old had better test scores. The ANOVA results showed that the frequency of participating in table tennis per week and the duration of each table tennis exercise had significant differences in the ATTS test score (all *p* < 0.01), and the more the participation in table tennis, the higher the score the participants get.

**FIGURE 1:**
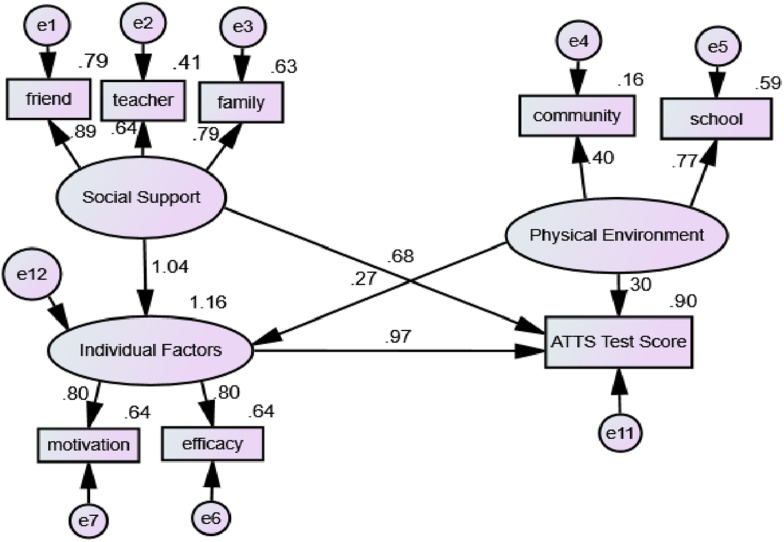
Final model of the variables. *Note*. friend: friends’ support; teacher: PE teachers’ support; parent: parents’ support; community: community environment; school: school environment.

Structural equation modeling was used to analyze the relationship between individual factors, social support, physical environment, and the ATTS test score. The results indicated that the structural model fit the data well (χ^2^ = 1415.754, χ^2^/df = 4.96, *p* < 0.001; CFI = 0.914; IFI = 0.930; NFI = 0.921; RMSEA = 0.032) (Figure 1). It is shown in Figure 1 that the individual factors, including self-efficacy and motivation, had a positive and significant effect on the test score (β = 0.97, *p* < 0.01). Social support from friends, PE teachers, and parents also had a significant effect on the ATTS test score (β = 0.68, *p* < 0.01). Furthermore, social support had an indirect effect on the ATTS test score through individual factors, and social support had a strong effect on individuals’ self-efficacy and motivation (β = 1.04, *p* < 0.01). The physical environment had a certain effect on the ATTS test score (β = 0.30, *p* < 0.01), and physical environment had an effect on adolescents’ motivation and self-efficacy (β = 0.27, *p* < 0.01).

## Discussion

In this study, it was found that: (1) individual factors played an important role for the improvement of the ATTS test score; (2) social support was positively related to the ATTS test score; and (3) physical environment was also significantly associated with the ATTS test score, which confirmed Hypothesis 1. The results of the multiple linear regression analysis and SEM showed that among the three levels, individual factors were the most important factors for the improvement of ATTS, which confirmed Hypothesis 2. Consistent with previous research evidence (Choo et al., 2015), self-efficacy was identified as an important factor related to the ATTS test score in this study. Similar results were also found by Kololo et al. (2012), whose study found that self-efficacy was a significant predictor of adolescents’ PA level. PA was a form of behavior that depended on an individual’s preference for activity type, frequency, duration, and intensity (Luke et al., 2004). Furthermore, PA in table tennis was positively related to the ATTS test score. Self-efficacy was significantly correlated with the persistence of individual participation in physical exercise and the frequency of exercise. This research indicated that motivation was highly correlated with ATTS test scores, which was consistent with the results of Wang and Zhang (2016). This study also showed that the ATTS test score was also affected by other individual factors such as age, sex, frequency, and duration of table tennis activities. Adolescents with more previous sports experiences (frequency, duration of table tennis activities) were easier to get a high ATTS test score (Condello et al., 2017).

The results of SEM illustrated that individual factors had a mediating effect between social support and the ATTS test score, which confirmed Hypothesis 3, and indicated that self-efficacy had been identified as a mediator between social support and PA. It was consistent with the study of McAuley et al. (2003). SEM also showed that individual factors served as a partial mediator of the effect that the physical environment had on the ATTS test score, which also confirmed Hypothesis 3. Moreover, physical environment had an effect on self-efficacy (Plotnikoff et al., 2010).

Another discovery of this research was that social support had a positive effect on adolescent sports activities, which was consistent with Wang’s (2017) finding. In the socio-ecological study of ATTS, social support was an inducement that could make adolescents perform better in table tennis. Support from parents and friends was more conducive to adolescents’ table tennis exercise than support from PE teachers. Adolescents’ exercise behavior was predicted by their intrinsic motivation (Gillison et al., 2006), and their opinion of PA was directly correlated to their exercise experience in PE lessons (Standage et al., 2012). There was a positive and supportive environment in PE, and their motivation and satisfaction with PA would be higher, which might form a virtuous circle and lead them to participate in exercise more frequently in school and leisure time (Ip et al., 2017).

In addition, it was proved in this study that physical environment had less influence on ATTS than individual factors and social support factors did. In China, the basic exercise locations for primary and secondary school students were schools and communities, and they spent more time to exercise with classmates in the school than in the community (Xie, 2018). However, due to the insufficient table tennis facilities in schools in Shanghai, there were few table tennis courses at schools (Yao, 2007). Similarly, communities also faced the problem of lacking of facilities (Wang et al., 2015; Zhu et al., 2015). Although physical environment had less influence on ATTS than individual factors and social support factors, the impact of the physical environment on ATTS could not be ignored. A favorable physical environment was closely related to students’ physical performance. Environments with PA-related resources, such as sidewalks, parks, sports classes, and health clubs, could make students more active in physical activities. The environmental lack of relevant resources or the presence of obstacles, such as bad weather or high crime rate, might reduce the students’ tendency to exercise (Loh et al., 2019).

The research of Yan et al. (2014) suggested that communities should provide sports infrastructure to support adolescents’ exercise. Schools should improve PE and offer more opportunities for spontaneous games (Langille and Rodgers, 2010). Communities and schools should work together to share resources for sports activities (Yan et al., 2014). Besides, our study revealed that students’ PA behaviors were affected by all factors of the social–ecological framework, which was consistent with Dollman’s findings (Dollman, 2018). James (2018) suggested that effective interventions and policies that aimed at improving PA enjoyment, availability of sports facilities, and social support from others should be prioritized to facilitate their PA persistence. Policy-makers should provide a wide “menu” of outdoor physical activities especially for table tennis from which students can choose, which was very important for establishing intrinsic motivation and PA persistence (Brug et al., 2017).

## Conclusion

The level impacting most significantly on ATTS is individual factors, followed by social support, and that with the least impact is the physical environment. Therefore, cultivating intrinsic interest is an important way to facilitate the continuous activities of adolescents. Second, friends should support each other, and parents should give adolescents appropriate encouragement regarding table tennis exercise. Schools should provide more table tennis facilities. PE teachers should respect adolescents’ ideas, listen to students’ opinions, and encourage them to participate in table tennis training.

## Limitations

The limitation of our study was that the subjects were from one metropolitan region, and the results might not be generalizable to other cities. Additionally, the policy level of social ecology was not covered in this study. The bivariate correlations between all the variables, including the sociodemographic variables, were not performed, and the sociodemographic variables were also not included in the model analysis. Future research can add policy factors to the study of ATTS and focus on other adolescents’ sports participation using the social–ecological model. Furthermore, it cannot be ignored that all the social–ecological measures were based on self-report, which may be interfered by the participants’ own perception of individual factors, social support, and physical environment. In addition, longitudinal studies and experimental research designs are necessary to further investigate factors that affect ATTS among Shanghai adolescents as the cross-sectional research design results may lead to difficulty in establishing cause and effect relationships among the study variables.

## Data Availability Statement

The datasets generated during the current study are not publicly available as they contain sensitive and potentially identifiable information. Requests to access these datasets should be directed to the corresponding author.

## Ethics Statement

Ethical approval for this study was obtained from the ethics committee at Shanghai University of Sport. All participants signed the written consent forms before they joined the study.

## Author Contributions

All authors contributed to this manuscript in performing literature search, conceptualizing, drafting, and revising the manuscript.

## Conflict of Interest

The authors declare that the research was conducted in the absence of any commercial or financial relationships that could be construed as a potential conflict of interest.

## Abbreviations

ATTS: adolescents’ table tennis skills
CFI: comparative fit index
IFI: incremental fit index
NFI: normed fit index
PE: physical education
RFI: relative fit index
RMSEA: root-mean-square error of approximation.
